# The impact of physical activity and intensity on clot mechanical microstructure and contraction in middle-aged/older habitual runners

**DOI:** 10.1186/s12883-025-04074-y

**Published:** 2025-03-01

**Authors:** J. C. Zaldua, O. Watson, D. J. Gregoire, S. Pillai, Y. Hellsten, K. Hawkins, P. A. Evans

**Affiliations:** 1 The Welsh Centre for Emergency Medicine Research, Emergency Department Morriston Hospital, Swansea Bay University Health Board, Swansea, Wales, SA6 6NL UK; 2 The August Krogh Section for Human Physiology, Department of Nutrition, Exercise and Sports, University of Copenhagen, Copenhagen, Denmark; 3 Faculty of Medicine and Life Health Sciences, Medical School, Swansea University, Swansea, SA6 6NL UK

**Keywords:** Clot microstructure, Clot mass, Clot contraction, Exercise, Physical activity, Middle-aged/olderadults, Endurance-trained, Runners

## Abstract

**Background:**

Exercise in healthy individuals is associated with a hypercoagulable phase, leading to a temporary increase in clot mass and strength, which are controlled by an effective fibrinolytic system. Conversely, people with cardiovascular diseases often have a reduced fibrinolytic pathway, increased clot mass and abnormal clot contraction, resulting in poorer outcomes. We assessed clot microstructure, particularly the contractile forces of clot formation, in response to two exercise intensities in middle-aged/older runners.

**Methods:**

Twenty-eight habitual male and female runners aged over 40 years completed a 10 km moderate-intensity run; 14 of them performed a 3 km high-intensity run. Blood samples were collected at baseline, immediately postexercise and after 1 h of rest. Clot structural biomarkers *d*_*f*,_ gel time, and measurements of mature clot mechanical properties (gel time, *G’*_*Max*_ and *CF*_*max*_) were analysed alongside conventional plasma markers.

**Results:**

Both exercise intensities altered markers of coagulant activity (PT, APTT and FVIII) and fibrinolysis (D-dimer), indicating hypercoagulability. Compared with longer-duration lower-intensity exercise, *d*_*f*_ was greater after short-duration intensified exercise bouts. Following an hour of rest, *d*_*f*_ dropped to baseline levels. Additionally, *CF*_*max*_ decreased across timepoints at both exercise intensities. This effect was noted after one hour of rest compared with baseline, suggesting continuous fibrinolytic activity postexercise.

**Conclusion:**

Exercise transiently induces an intensity-dependent hypercoagulable state, resulting in denser clot formation and a reduced clot contractile force due to fibrinolysis. These findings can help guide the safe commencement of rehabilitation exercise programs for cerebrovascular patients.

**Supplementary Information:**

The online version contains supplementary material available at 10.1186/s12883-025-04074-y.

## Highlights

Precise rheological techniques were applied to assess the effects of exercise on clot microstructure (*d*_*f*_) and clot contraction in middle-aged/older-aged men and women.

The hypercoagulable phase, as determined by plasma markers and *d*_*f*_, increased after intense exercise.

Clot contractile forces (*CF*_*max*_) were reduced one hour after moderate and intense exercise, indicating enhanced fibrinolysis.

We propose that *d*_*f*_*and CF*_*max*_ may serve as indicators of health risks and benefits of exercise in patients undergoing exercise rehabilitation after undergoing adverse cardiovascular events.

## Introduction

A normal hemostatic system is sustained by a delicate balance between thrombotic and fibrinolytic processes. The resulting activity of the procoagulant phases, when activated, leads to the initial formation of clots. This biomechanical structure is modified and maintained by the balancing effect of the fibrinolytic system. Furthermore, clot stabilisation and strengthening occur through platelets pulling fibrin and cross-linking, resulting in a mature clot that subsequently contracts.

Increased blood flow and shear rates associated with exercise are known to influence platelet activity and clotting factors, leading to a recognised temporary hypercoagulable phase that returns to baseline levels postexercise. In trained adults, the regulation of fibrinolysis [1–4] and a healthy functional vascular endothelial system [5] contribute to an even more pronounced and positive effect on maintaining hemostasis. Recent studies have shown that exercise promotes hypercoagulability in young and untrained individuals, resulting in increased clot formation and density, and that the impact of exercise increases with intensity [6, 7]. However, few studies have utilised exercise as an intervention that determines the impact of clot mechanical development and the contractile phase in middleaged/olderadults.

The hypercoagulable phase directly influences the templating of the primary structure of the incipient clot (*df*), which is known to define its mature architecture [8, 9]. The newly formed clot produced during the early phase of coagulation develops a cross-linked fibrin network. A denser, more tightly packed fibrin network composed of thinner fibres is far less susceptible to lysis and may enhance thrombogenicity; a loose, open, and permeable fibrin network allows lysis enzymes to degrade blood clots more easily. This information can be quantified through the rheological biomarker fractal dimension (*d*_*f*_), which quantifies the “sample-spanning” network of a fractal structure [10].

The incipient clot network, as characterised by *d*_*f*_, provides a microstructural template for the mature form of the clot. This biomarker has been used to quantify the thrombotic potential of a range of conditions, including stroke and ischaemic heart disease [11–15], and to evaluate the secondary effects of clinical treatments [16, 17]. This clot microstructure biomarker has also been utilised to assess the influence of a period of exercise training on the hypercoagulable phase in healthy middleaged/older female participants and has demonstrated a significant effect [18, 19]. Interestingly, in a recent study examining the effect of acute exercise and its intensity in poststroke patients, researchers reported a significant increase in *d*_*f*_ after exercise [20]. However, the investigation did not ascertain the effects of the contractile properties of the clot.

Clot contraction may provide crucial insights into pathological development, the quality of the clot and its altered functionality, especially in determining how the mature clot decreases in size over time, which aids in the recanalisation of blood vessels and prevents further thrombus formation [21]. However, this effect has mostly been evaluated via nonhemorheological methods. The paucity of evidence on the mechanical properties of the developing clot and its associated clot contraction during exercise has proven to be a worthwhile area of investigation. Furthermore, altered contractile forces in clot development have been noted to be important factors in abnormal clot formation in vascular diseases [22, 23].

Our aim was to determine how exercise at moderate or more intensive exercise affects the microstructure of an incipient clot (*d*_*f*_*)* and the maximum contractile force (*CF*_*max*_) of the mature clot in a cohort of well-trained, middle and older-aged runners.

## Materials and methods

### Healthy participant recruitment

We recruited people above 40 years of age from local running clubs who regularly engage in endurance exercise, where self reported regular exercise frequency was at least 3-4x per week, and for whom a 10 km run could be completed with difficulty, Two-stage informed consent was obtained following proper health screening. They were screened for acute and chronic medical conditions, particularly relating to cardiovascular health using a healthy volunteer questionnaire delivered by trained clinicians. Individuals reporting these conditions or taking regular medications were not invited to participate.

The choice of a cohort upwards of 40 without an upper age limit was a pragmatic approach to recruit a cohort of an appropriate age to draw conclusions on thrombotic diseases which become increasingly prevalent in the population with age. There is no widely accepted scientific distinction between middle and older-age and a recognition of the transition between these groups is dependent on an array of health, socio-economic and cultural factors. Within this study, a lower age cut off for inclusion was necessary, whilst other inclusion criteria of good health and regular physical exercise provided a self-selecting upper age limit on the study.

The study was divided into two arms: the longer 10 km at moderate intensity (LMI) arms and the shorter 3 km at a higher intensity (SHI) arms. For the LMI study arm, participants ran at a steady self-selected pace along a flat 10 km route. Following completion of the LMI arm of the study, all participants were re-invited back to complete the SHI arm until the necessary number of participants was obtained. There were no additional inclusion or exclusion criteria for re-invitation to the SHI arm once the LMI arm was completed. For the SHI arm, participants ran 3 km around a 400 m athletics track as fast as possible, with the option to perform a self-selected warm-up routine beforehand. Lactate levels taken at each time point were used to confirm the intensity of the self selected paces and ensure adequate distinction in the intensities between the two arms [24, 25].

### Sample collection and timing

Blood samples were taken by a trained phlebotomist atraumatically from an antecubital vein via a 21G butterfly needle at baseline, immediately following exercise, and after 1 h of rest. To avoid the effects of diurnal variation, the experiments were conducted in the morning, and all samples were obtained before 12 p.m.

### Conventional markers of coagulation and full blood count

We measured standard full blood counts and markers of coagulation, full blood count (FBC), prothrombin time (PT), activated partial thromboplastin time (aPTT), D-dimer, and factor VIII (FVIII).

For FBC, 4 mL was collected in a plastic dipotassium EDTA vacuette (BD, Plymouth, UK Ref 367839) analysed in Sysmex XN9000.

For standard coagulation tests, two 2.7 mL samples were collected into PET 0.109 M 3.2% citrated vacutainers (BD, Plymouth, UK Ref: 363095). The first citrated vacutainer included PT, APTT, and Clauss fibrinogen and was measured via a Sysmex CS5100 analyser with reagents for PT-Siemens Innovin, APTT- Siemens Actin FS and fibrinogen- Siemens thrombin. D-dimer analysis was performed with a Sysmex CS5100. The second citrated sample was used for FVIII analysis and placed in a centrifuge using an Eppendorf 5427R + FA-45-12-17 Rotor at 2000 × g for 10 min to obtain platelet-poor plasma (PPP).

### *Lactate* measurement

A total of 2.7 mL of venous blood was collected into a BD Vacutainer REF 368,921 at each time point, and the lactate concentration was analysed via the Roche assay in Cobas 8000.

### Rheological technique

8 ml. Whole unadulterated blood was collected to measure the time to gel point (T_*GP*_*)*, fractal dimension (*d*_*f*_*)*, maximum value of G’ (G’_max_) and maximum contractile force (*CF*_*max*_). The data were anonymised and independently reviewed as per the STARD and STROBE guidelines.

The rheological measurements were performed via small-amplitude oscillatory shear measurements at several discrete frequencies [10]. Approximately 6.7 ml of blood was transferred to a double concentric measuring geometry of an AR-G2 (TA Instruments, New Castle, DE, USA), controlled at a temperature of 37 °C. A negligible quantity of standard silicon oil (10 mPa.s) was placed around the edge of the geometry to prevent evaporation of the sample. The phase angle (i.e., the lag in phase between the stress and strain waveforms) was monitored during clotting. The gel point was detected by the frequency independence of the phase angle, and the time to reach this point was recorded as the time to the gel point (T_*GP*_). The fractal dimension (*d*_*f*_) was calculated from an established mathematical relationship at Gel Point [26] and provides quantification of the incipient clot microstructure [10].

*CF*_*max*_ is calculated from rheological measurements of normal force and represents a measurement of the contractile forces generated by blood during clotting [21, 22, 27]. As clotting progresses, the matrix scaffold interlinked by the fibrin network, platelets and other components generates and applies forces to the surrounding microstructure and the rheometer geometry. Normal force measurements were conducted via the parallel plate geometry (60 mm diameter) of a second AR-G2 rheometer, with the lower plate controlled at 37°C. Approximately 0.84 ml of the same sample of blood was loaded onto the lower plate of the rheometer, and the upper plate was gradually lowered to confine the sample between the plates at a fixed gap of 300 microns. Standard 10 mPa.s silicon oil (Brookfield) was again placed around the edge of the geometry to prevent evaporation of the sample. The measurements involved the application of small-amplitude oscillatory shear at a single frequency of 1 Hz. The normal force generated between the two plates was recorded over time by the normal force transducer fitted to the lower plate. *CF*_*max*_ was defined herein as the difference between the value of the normal force at the instant corresponding to the gel point and the maximum value of the normal force registered during clot formation. This ensured that *CF*_*max*_ was attributable to the forces exerted on the fibrin network that was established at the gel point. The test ran for 75 minutes to record the maximum value of *G’*, which is representative of the elasticity of the fully formed clot (*G’*_*max*_), and the value of *CF*_*max*_ generated by the clot.

### Statistical analysis

IBM SPSS Statistics 29.0.1.0.171 was utilised to perform the statistical analysis. To estimate the necessary sample sizes, the alpha level was set to 0.05 with a power level of 0.85 on the basis of previous reports measuring fractal dimension [6, 10, 12, 13, 28]. The sample size needed was 10 for each additional time point. For attrition, we recruited 28 endurance runners aged over 40 years for the LMI group, and 14 were invited to return for the SHI arm of the study.

The normality of the data was confirmed via the Shapiro‒Wilk test. Outliers were detected via boxplot analysis in IBM SPSS; however, none were excluded, as this was not justified from either a clinical or a technical standpoint. Statistical significance was accepted at *p* ≤ 0.05. Normally distributed data are reported as the mean (*M*) and standard deviation (*SD*) and are displayed in figures as bar charts with uncertainty bars, whereas nonnormally distributed data are shown as the median (*Mdn*) and interquartile range (*IQR*) and are presented as box and whisker plots.

All the statistical analyses were performed using repeated-measures analysis of variance (RM-ANOVA) with a Bonferroni post hoc correction for normally distributed data sets involving three time points and the Friedman test for nonnormally distributed data followed by a nonparametric Wilcoxon matched-pairs signed rank test with a Bonferroni adjustment level set at *p* < 0.016, which helps correct for type I error for any variables with a significance value of *p* < 0.05. For RM-ANOVA, if the assumption of sphericity was violated, the Greenhouse–Geisser correction *p* value was accepted for interpretation.

To compare individual variables between groups, paired t tests were carried out for normally distributed data, and the Wilcoxon matched-pairs signed rank test was used as the nonparametric counterpart. The difference scores were assessed for distributional assumptions. If the data were not met, appropriate data transformations or sign tests were utilised. Figures were created via GraphPad Prism (version 9.3.1) (Tables 1 and 2).

## Results

**Table 1:** Basic demographic data of the study participants

Demographic	LMI (10 km) *n* = 28	SHI (3 km) *n* = 14
Age	57 ± 8	60 ± 6
Sex	13 Female: 15 Male	6 Female: 8 Male
Height (cm)	168.9 ± 9.5	166.9 ± 12.8
Weight (kg)	68.2 ± 13.5	66.9 ± 12.9
BMI	23.5 ± 3	23.7 ± 2.2
Pace (mm: ss)	6:00 ± 60 s	4:57 ± 45 s

Note: Each participant habitually runs three to six times weekly. Pace was recorded as the average time taken to complete each kilometer (minutes: seconds/km). Pace during LMI for the participants that also conducted SHI was 06:05 ± 65 s

**Table 2:** Selected paired comparisons of the LMI and SHI intensity groups

Measured parameter	Long moderate intensity		Short high intensity		Test statistics	Significance value (*p*)
	M/Mdn	SD/IQR	M/Mdn	SD/IQR		
PT	10.5	IQR 0.75	10.5	IQR 0.42	z=-5.37	p 0.59
APTT	25	± 1.59	25.5	± 1.49	t(11)= -1.4	p 0.1
Fibrinogen	2.9	± 0.46	2.88	± 0.42	t(11) = 0.18	p 0.86
WBC	7.4	± 2	7.93	± 2.3	*t*(13)= -1.01	*p* 0.3
Hb	145.7	± 8.97	144.5	± 10.02	*t(*13) = 0.87	*p* 0.4
Platelets	289	*IQR* 63	279	*IQR* 54	*z*= -0.88	*p* 0.38
RBC	4.8	± 0.37	4.8	± 0.38	*t*(13) = 0.28	*p* 0.79
HCT	0.4	± 0.02	0.44	± 0.26	*t*(13) = -2.2	*p* 0.05

### Standard blood and coagulation markers

Hb, Hct and Rbc slightly but significantly decreased after both exercise bouts, with a marked decrease after a shorter run at higher intensity. Statistically significant, but clinically non-relevant increases in WBC were seen in both groups following exercise, while the platelet count returned to baseline levels after exercise activity.

Although PT and APTT remained within their normal ranges, exercise resulted in an inverse trend compared with baseline; PT increased, whereas APTT slightly decreased. The effect was more significantly different for SHI, and the increasing hypercoagulable phase even lasted after 1 h of rest postexercise. Despite these statistically significant values, no clinical interpretation can be derived, as they remain within the normal range. Figure 5 (supplementary section) depicts these changes.

The levels of FVIII and D-dimer in both groups were the only variables that exceeded their preestablished clinical normal ranges after exercise. D-dimer levels were greater in both groups immediately after exercise than at baseline (LMI: *Mdn* 200 µg/L vs. 335 µg/L, *p* = < 0.001; SHI: *Mdn* 202 µg/L vs. 560 µg/L, *p* = 0.01), and the levels returned to baseline levels after 1 h of rest (LMI: *Mdn* 200 µg/L vs. 236 µg/L, *p* = 0.05; SHI: *Mdn* 202 µg/L vs. 231 µg/L, *p* = 0.13).

The factor VIII levels increased from baseline to after exercise in both groups (LMI *Mdn* 129 iu/dl vs. 171 iu/dl, *p* = 0.012; SHI *Mdn* 128 iu/dL vs. 202 iu/dL, *p* = < 0.001) and remained elevated after an hour of rest compared with the baseline data (LMI, *Mdn* 129 iu/dl vs. 156 iu/dl, *p* = 0.01; SHI *Mdn* 128 iu/dl vs. 187 iu/dl, *p* = < 0.001). Figure 6 in the supplementary section demonstrates these changes.

Both the factor VIII (*p* < 0.01) and D-dimer (*p* < 0.05) levels were significantly greater in the SHI group immediately after exercise. Figure 7 in the supplementary section illustrates these changes.

### Lactate levels

**Fig. 1:**
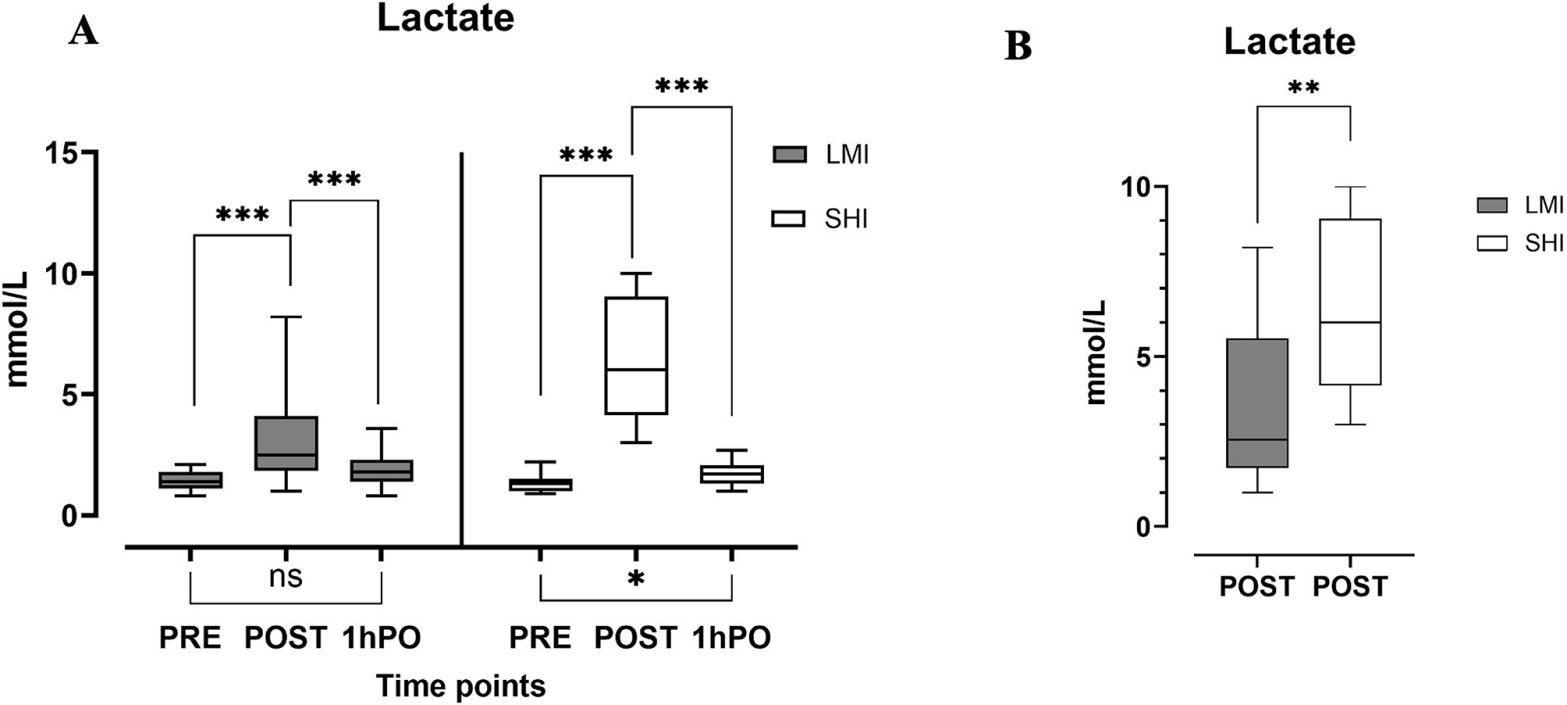
Lactate levels before and after LMI and SHI. *Note. (left)*** A** Lactate levels across three time points. *(right)*** B** Paired comparison of lactate levels measured immediately after exercise at LMI and SHI exercise intensities. PRE (before exercise), POST (immediately after exercise), and 1hPO (1 h after rest) were used. Error bars (IQRs) for median values; *p < 0.05, **p < 0.01, and *** *p* < 0.001 denote the statistical level; ns, not significant

The blood lactate levels measured at each time point are shown in Fig. 1a and b. In the LMI group, lactate levels increased (*p = <* 0.001) from baseline to after exercise, and the levels returned to baseline at 1 h of rest. In the SHI group, there was an increase (*p* = < 0.001) from baseline to after exercise, and the level remained elevated at 1 h of rest (*p* = < 0.001). Moreover, a direct comparison of the lactate response in the subgroup of 14 participants who underwent both SHI and LMI revealed a statistically significant difference in lactate levels after exercise between the two bouts (Fig. 1b; *p* = 0.008).

### Hemorheological data

**Fig. 2:**
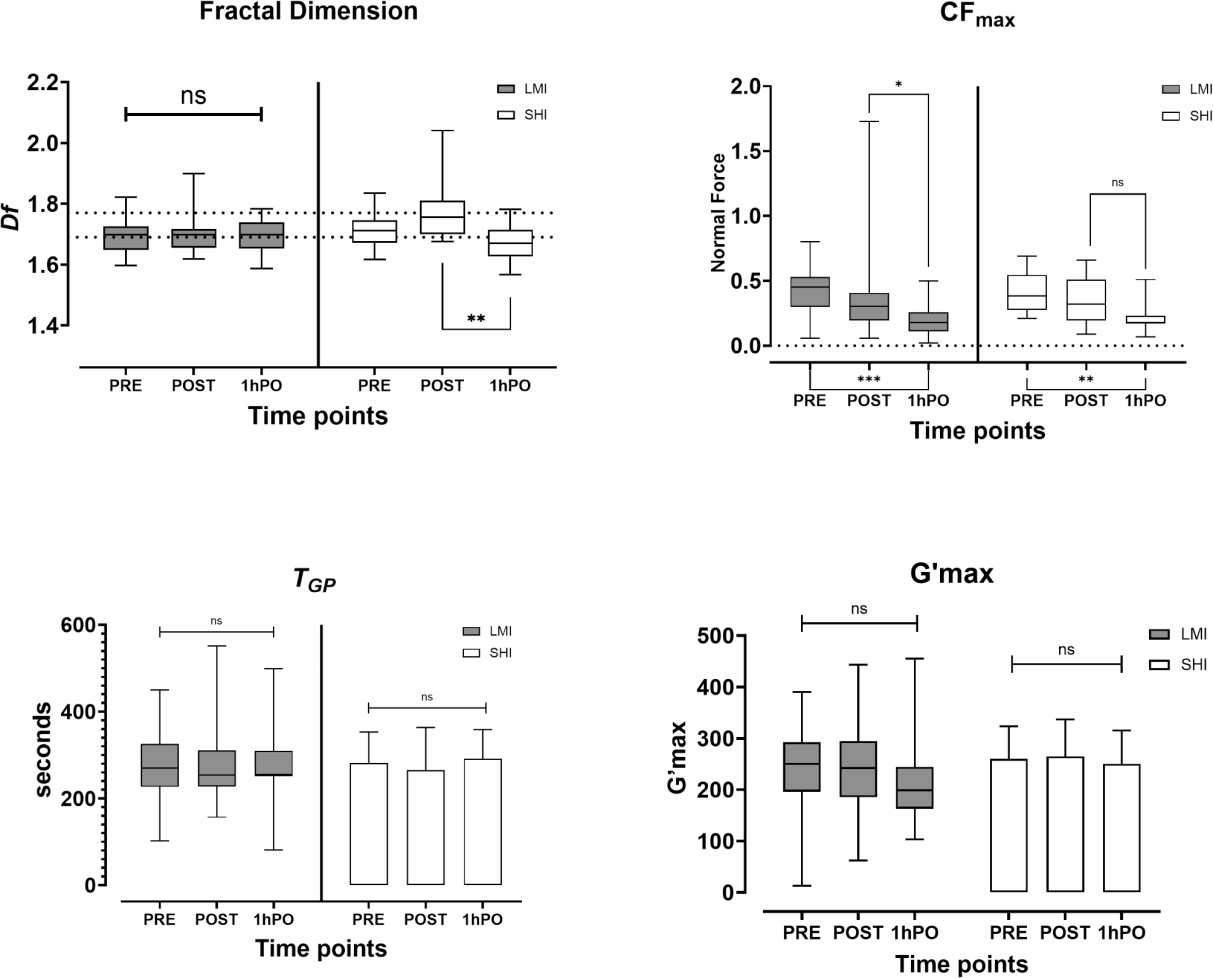
Hemorheological markers of the LMI and SHI intensity groups at three time points. Note. PRE (before exercise), POST (immediately after exercise), and 1hPO (1 h after rest) were used. The dashed line on the y-axis depicts the upper and lower ranges. The error bars represent the SDs for the means and IQRs for the median values; *p < 0.05, **p < 0.01, and *** *p* < 0.001 denote the statistical level; ns, not significant

The results for the LMI and SHI are shown in Fig. 2. In the LMI group, there were no significant changes in *d*_*f*_ between any of the time points. In the SHI group, there were no significant changes in *d*_*f*_ between baseline and immediately after exercise; however, a significant reduction in *d*_*f*_ was observed between immediately after exercise and after 1 h of rest (*Mdn* 1.74 vs. 1.66, *p* = 0.003).

Both groups exhibited an overall reduction (main effect) in *CF*_*max*_ with time. In the LMI group, there was no difference in the *CF*_*max*_ between baseline and immediately after exercise (*p* = 0.2). Conversely, the *CF*_*max*_*values* immediately postexercise and after 1 h of rest were significantly lower (*Mdn* 0.30 vs. 0.19, *p* = 0.014). Overall, a significant decrease was observed in the *CF*_*max*_ between baseline and after 1 h of rest in the LMI group (*Mdn* 0.42 vs. 0.19, *p* = 0.002). In the SHI group, there was no difference in the *CF*_*max*_ between baseline and immediately after exercise (*p* = 0.5), and there was no significant difference between immediately after exercise and after one hour of rest (*p* = 0.15). However, there was a significant reduction in the *CF*_*max*_ from baseline to after 1 h of rest (*Mdn* 0.36 vs. 0.18, *p* = < 0.001).

### Paired comparisons of hemorheological data

**Fig. 3:**
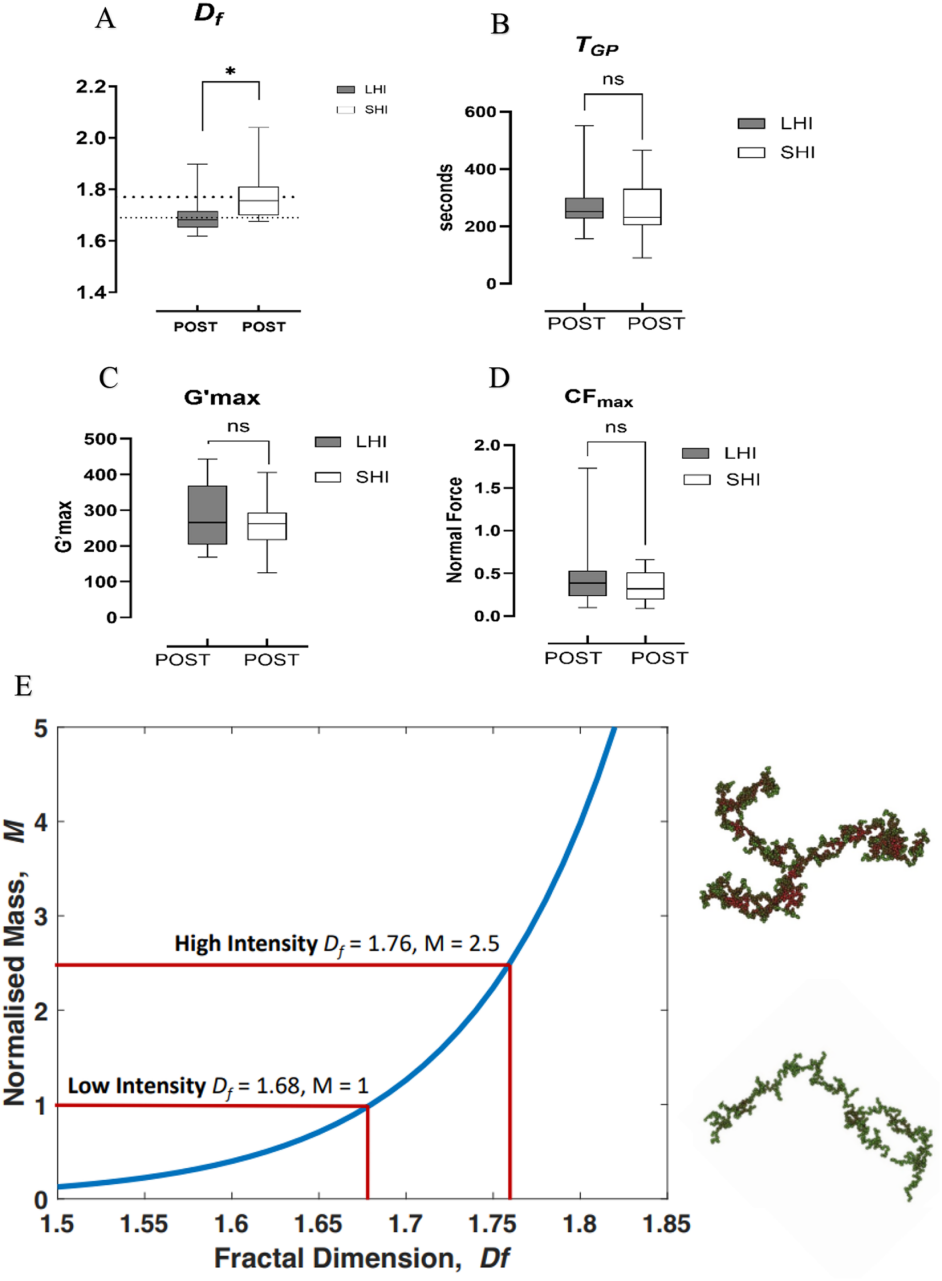
Paired comparison of (**A**) d_f_, (**B**) T_GP_, (**C**) G’_max_, (**D**) CF_max_ and (**E**) relative clot mass M based on values of fractal dimension in the LMI and SHI at immediately following exercise. Note. The dashed line on the y-axis depicts the upper and lower ranges. The error bars represent the IQRs for median values; *p < 0.05 denotes the statistical level. (**E**) Connected fibrin fibres are depicted by green and red dots, with red dots corresponding to areas of maximum density reflecting a more complex interconnected microstructure (i.e., greater mass). In the SHI group and its hypercoagulable phase, there were 2.5 times more clots than clots in the LMI group. This is illustrated by the nonlinear relationship between d_f_ and the clot mass (for illustrative purposes, the clot mass has been normalised to a value of 1 at the mean d_f_ value of the LMI group)

The haemorheology results comparing values obtained immediately after exercise in the LMI and SHI runs are displayed in Fig. 3. *d*_*f*_ immediately after exercise was significantly lower in the LMI group than in the SHI group (*Mdn* 1.68 vs. 1.76, *p* = 0.02), whereas *CF*_*max*_, *G’*_*max*_ and *T*_*GP*_ were not significantly different. The relationship between *d*_*f*_ and the total amount of fibrin mass incorporated into the clot structure is nonlinear, and relatively small changes in the absolute value of *d*_*f*_ reflect large differences in clot mass. Computationally simulated fractal networks (Adapted from Lawrence et al. 2015, reproduced with permission from Elsevier) depicting the increase in incipient clot mass corresponding to increases in *d*_*f*_ are shown in Fig. 4E [14]. The observed difference of 1.68 after the 10 km run compared with 1.76 after the more intense 3 km run represents a 250% difference in clot mass.

## Discussion

The present study used exercise intensity to assess the hypercoagulable effect and changes in clot microstructure in habitually trained middle-aged/older male and female runners. While *d*_*f*_ remained stable in both immediately post-exercise bouts, a more hypercoagulable phase was detected after shorter, more intense exercise was compared to longer duration, less intensive exercise in the same participants. The hypercoagulability was transient, returning to baseline within an hour of rest. Additionally, there was a statistically significant decrease between immediately post and after 1 h rest in LMI, this change was non-significant post-hoc correction when compared to its baseline values. Primarily, this effect may be due to the release of plasminogen as a physiological fibrinolytic response during an increase physical activity which makes the clot’s fibrin network less strong as measured by the *df*. Our study validated previous findings that *d*_*f*_ accurately measures the alteration of clot microstructure in healthy and trained athletes [18]. Interestingly, untrained participants in a previous study achieved higher *d*_*f*_ levels as a result of intense exercise than older trained athletes [6]. This study supports the idea that higher exercise intensities lead to a temporary alteration in clot microstructure and density.

### Fractal dimension (*df)*

The trained middle-aged/olderolder participants had a lower post-exercise level of *d*_*f*_ than the young and untrained participants (1.70 ± 0.07 vs. 1.79 ± 0.05) [6], primarily because of their improved hemostatic profile [29–31]. This improvement is partly due to the marked effect of endurance training on endothelial function, leading to increased production of nitric oxide (NO) [32, 33], a potent vasodilator and anti-inflammatory compound. It also increases the capacity for the formation of prostacyclin, a strong vasodilator and an important inhibitor of platelets [34]. The vasodilatory effects of NO and prostacyclin lead to a reduction in intravascular shear stress when combined with their platelet inhibitory effects, effectively decreasing platelet‒vessel wall contact [35, 36]. This leads to dampening of the hypercoagulable phase and reduces the risk of thrombosis. The evidence suggests that, with as little as one month of endurance training, this response can be sufficiently achieved [37]. Furthermore, compared with sedentary runners, endurance runners present a reduced level of von Willebrand factor (vWf) antigen activity [38] and decreased platelet aggregation [39].

Another factor that could contribute to this effect is the efficiency and effectiveness of the fibrinolytic system. This is supported by a stable df, a reduction in CFmax and increased D-dimer levels, which returned to baseline. Fibrinolysis occurs simultaneously alongside the primary development of the fibrin network in the vasculature [40]. Similarly, acute endurance exercise not only generates an immediate fibrinolytic response [4, 41, 42] but also enhances fibrinolysis in the long-term [1–4]. A recent study demonstrated that postmenopausal women who had recently completed a structured exercise training program for more than 8 weeks had a 40% reduction in incorporated clot mass following the training period [18]. Individuals adapted to physical activity are known to have heightened resting fibrinolytic activity, which is attributed to increased tPA release, decreased PAI-1 activity and decreased tPA-PAI-1 complex formation [1, 4]. Our data support this finding in showing that df was relatively stable across the three time points at both exercise intensities. However, when a paired comparison was made, post-exercise df was greater in SHI than in LMI. As higher-intensity exercise is associated with an increase in shear and blood flow, these factors might have surpassed the effect of increased basal fibrinolytic activity

In a previous exercise study involving young, untrained participants, *d*_*f*_ was observed to significantly increase during the duration of exercise activity before returning to baseline after a period of rest [6]. This finding showed that moderate-intensity exercise was sufficient to lead to an increase in *d*_*f*_. While our results revealed a higher level of *d*_*f*_ after a higher exercise intensity, a sequential comparison of *d*_*f*_ values in two separate bouts revealed that *d*_*f*_ remained stable over time. As other studies have demonstrated that the fibrinolytic response can vary with fitness level [43] we suggest that hemostatic adaptations caused by exercise between our trained group and the young sedentary group can explain the differing responses to these exercise bouts.

### G’max and TGP

*G’*_*max*_ and *T*_*GP*_ remained stable at both intensities despite the hypercoagulable phase and fibrinolytic activity, as shown by changes in *d*_*f*_ and *CF*_*max*_, respectively. This finding contrasts with previous findings using ROTEM, in which maximum clot firmness (MCF) has been shown to increase with exercise [44, 45]. Notably, *d*_*f*_ is the only structural marker, whereas *G’*_*max*_ reflects the mechanical properties of the mature clot, and *T*_*GP*_ is a kinetic marker of clot formation. The different findings from our earlier study of untrained yet healthy young participants showed that a more marked response to the hypercoagulable phase corresponds to increasing clot mass and intensity. This response is normalised within an hour [6]The significance of these findings is that they might in future be used to develop and guide exercise intensity targets for patients embarking on an exercise programme as part of a structured exercise prescription such as those used following conditions such as stroke or myocardial infarction (MI).

### Standard blood, coagulation markers and lactate response to exercise bouts

We hypothesize that the platelet response is likely due to recognised exercise-induced haemoconcentration [46] and increasing intensity. Although there were significant changes in Hb, Hct and Rbc across timepoints, the levels remained within their clinically assigned normal ranges, as expected given that the participants were physically active and healthy. Figure 4 (supplementary section) provides a detailed view. In SHI only, fibrinogen was markedly lower after 1 h of rest. The primarily reason for this could be due to volume changes [47], chronic adaptation [48] and initial utilisation of fibrinogen in clot formation, as reflected by increasing *d*_*f*_ immediately postexercise. The marked increase in FVIII and D-dimer with increasing exercise intensity indicates increased thrombotic and fibrinolytic activity.As seen in Fig. 1, there was a significant difference in the exercise intensities achieved in both arms of the study, While a small degree of overlap was seen, no individual participant had a higher lactate level in the LMI arm than in the SHI arm. Additionally participants all had a faster pace and higher rate of perceived exertion (RPE) in the SHI arm compared with the LMI arm. More complex intensity validation procedures are possible, including real time analysis of RPE, lactate via point of care testing, or heart rate. However the focus of these exercise targets is implementation of large scale exercise prescriptions, often these take place with initial supervision, followed by transition to unsupervised or non-clinician supervised activities. The focus in these programmes is to provide simple recommendations deliverable at scale, as such more complex intensity validation techniques may be less appropriate.

### The effects of exercise and its hypercoagulable phase on clot contractile forces

There was a significant reduction in the *CF*_*max*_ following exercise. A lower level of *CF*_*max*_ accompanied by a normal *d*_*f*_ may suggest a less dense fibrin network, which may allow greater accessibility of fibrinolytic agents. This effect appeared to persist longer than the other measured markers, as *CF*_*max*_ remained low even after an hour of rest.

Previous studies using different techniques have suggested that clot contraction is reduced in patients who suffer a stroke and that this poor contraction is associated with a worse outcome [49]. In our cohort study, which used a different technique, we showed that the clot contractile forces were also reduced with increasing intensity exercise under the influence of a hypercoagulable phase, as measured by the *CF*_*max*_. The reason for this may be that the participants in our study may have had a highly effective and conditioned fibrinolytic system [1, 4, 38, 50, 51], which reduces the contractile forces, leading to a normal physiological process and reduced thrombogenicity. However, patients who have suffered a stroke are known to have an enhanced prothrombotic pathway and an altered fibrinolytic system, which in turn alters the mechanical properties of the clot microstructure, producing an obstructive thrombus. In our previous study, we examined the effects of exercise and intensity on lacunar stroke patients and confirmed that exercise transiently increased *d*_*f*_ levels [20]. Compared with the control group, the physically inactive group was found to have greater thrombogenic risk posed by acute moderate exercise. However, this research did not examine the relationship between the templating effect of *d*_*f*_ and its associated clot contractile forces.

The prolonged reduction in clot contraction after one hour of rest is likely due to factors affecting clot initiation and fibrinolysis. Compared with untrained healthy participants, trained healthy participants exhibit reduced platelet activity [39]. This, combined with the transient effect of thrombin generation, which leads to short-lived hypercoagulability following exercise [52], may have contributed to the reduced *CF*_*max*_. Conversely, the fibrinolytic activity induced by exercise in trained individuals is known to last up to 24 h [53, 54]. This may account for the long-term effect of exercise in reducing the amount of clot contractile mechanical process and its functional activity.

Hence, fibrinolysis is likely involved in the prolonged reduction in clot contraction. While clot degradation products such as D-dimer return to baseline levels after one hour of rest, trained individuals’ endogenous fibrinolytic activity can last longer [53, 54]. The effective breakdown of clots immediately after exercise through known adaptive levels of lytic enzymes such as t-PA and PAI-1 [38] may effectively lyse fibrin and dampen the overall contractility of the clot. This may act as a protective component against cardiovascular and cerebrovascular diseases in trained individuals because of more effective clot dissolution. Ultimately, the novelty of these findings lies in the accurate measurement of the clot contractile force, leading to the assessment of the fibrinolytic response following exercise. Understanding the contributing factors for such findings requires thorough and in vitro investigations, which are underway.

## Summary and conclusion

We describe for the first time the utility of *d*_*f*_ and *CF*_*max*_ throughout the hypercoagulable phase associated with exercise in trained middle and older-aged runners. Additionally, *d*_*f*_ showed sensitivity to exercise intensity in this trained cohort. It remains to be seen if these findings could be translated to clinical practice to identify patients with high thrombotic risk when a new exercise program is commenced. It may be applied to those undergoing a rehabilitation programme following conditions such as stroke or MI. Furthermore, the ability to measure the effects of hypercoagulable changes and the clot contractile force via these biomarkers in whole blood during near-patient testing can increase its diagnostic potential in hypercoagulable states.

We propose that *d*_*f*_ and *CF*_*max*_ are valuable biomarkers that can be used to guide safe commencement of exercise, which may help determine the appropriate intensity thresholds throughout exercise progression in different populations. The biomarkers may be specifically useful in patients with cerebrovascular disease undergoing rehabilitation programs of varying intensities and who might be at risk of developing adverse cerebrovascular events. Further work is underway to further determine the applicability of these biomarkers in relation to exercise in this disease group.

## Electronic supplementary material

Below is the link to the electronic supplementary material.

Supplementary Material 1

## Data Availability

The data sets used and/or analysed during the current study are available from the corresponding author upon reasonable request.
